# Genome-Wide Analysis of *Myeloblastosis-Related* Genes in *Brassica napus* L. and Positive Modulation of Osmotic Tolerance by *BnMRD107*

**DOI:** 10.3389/fpls.2021.678202

**Published:** 2021-06-17

**Authors:** Jian Li, Keyun Lin, Shuai Zhang, Jian Wu, Yujie Fang, Youping Wang

**Affiliations:** ^1^Key Laboratory of Plant Functional Genomics of the Ministry of Education/Jiangsu Key Laboratory of Crop Genomics and Molecular Breeding, Yangzhou University, Yangzhou, China; ^2^Joint International Research Laboratory of Agriculture and Agri-Product Safety, The Ministry of Education of China, Yangzhou University, Yangzhou, China

**Keywords:** *Brassica napus*, MYB-related transcription factors, phylogenetic analysis, expression profiles, abiotic stress

## Abstract

Myeloblastosis (MYB)-related transcription factors comprise a large subfamily of the MYB family. They play significant roles in plant development and in stress responses. However, MYB-related proteins have not been comprehensively investigated in rapeseed (*Brassica napus* L.). In the present study, a genome-wide analysis of MYB-related transcription factors was performed in rapeseed. We identified 251 *Brassica napus MYB* (BnMYB)-related members, which were divided phylogenetically into five clades. Evolutionary analysis suggested that whole genome duplication and segmental duplication events have played a significant role in the expansion of *BnMYB-related* gene family. Selective pressure of *BnMYB-related* genes was estimated using the *Ka/Ks* ratio, which indicated that *BnMYB-related* genes underwent strong purifying selection during evolution. *In silico* analysis showed that various development-associated, phytohormone-responsive, and stress-related *cis*-acting regulatory elements were enriched in the promoter regions of *BnMYB-related* genes. Furthermore, *MYB-related* genes with tissue or organ-specific, stress-responsive expression patterns were identified in *B. napus* based on temporospatial and abiotic stress expression profiles. Among the stress-responsive *MYB-related* genes, *BnMRD107* was strongly induced by drought stress, and was therefore selected for functional study. Rapeseed seedlings overexpressing *BnMRD107* showed improved resistance to osmotic stress. Our findings not only lay a foundation for further functional characterization of *BnMYB-related* genes, but also provide valuable clues to determine candidate genes for future genetic improvement of *B. napus*.

## Introduction

Transcription factors play a major role in the regulation of gene expression. Myeloblastosis (MYB) proteins represent a large family of transcription factors, members of which perform diverse biological functions in all eukaryotes. MYB transcription factors are characterized by a conserved MYB domain containing one to four imperfect repeats of ~52 amino acids (Dubos et al., 2010). Generally, each repeat can form three alpha helices, among which the second and third helices shape the helix-turn-helix structure *via* three consistently spaced tryptophan residues, which are essential to form the hydrophobic core of the amino acids, thereby stabilizing the three-dimensional structure of MYB repeat and promoting DNA-binding (Sue et al., 2006). In plants, MYB transcription factors can be roughly divided into four categories, designated as 1R-MYB, R2R3-MYB (2R-MYB), R1R2R3-MYB (3R-MYB), and 4R-MYB, according to the number of adjacent repeats (R) in MYB domain, which is located at the N-terminus (Dubos et al., 2010). R2R3-MYB is the predominant subgroup, with multiple regulatory functions covering almost every aspect of plant life-cycle, such as cell shape determination, secondary metabolism, plant organ patterning, and stress responses (Aydin et al., 2014). To date, studies have mostly concentrated on R2R3-MYB genes because of the large number of members of this subfamily. R1R2R3-MYB transcription factors bind to mitosis-specific activator (MSA) element, and modulate cytokinesis and cell cycle in plants (Haga et al., 2007). R4-type MYB proteins were identified later in plants, and little is known about their functions (Chen et al., 2006). 1R-MYB type proteins, also known as the MYB-related transcription factors, usually contain a single (sometimes only partial) binding repeat (Kirik and Baumlein, 1996; Li et al., 2019). MYB-related proteins act as pivotal regulatory components by activating or repressing gene transcription in plants (Andronis et al., 2008).

The first *MYB-related* gene (*mybSt1*) in plants was identified in potato and was demonstrated to function as a transcriptional activator (Baranowskij et al., 1994). In recent decades, a growing number of *MYB-related* genes were subsequently characterized in many plant species, including *Arabidopsis thaliana* (Yong et al., 2019), *Oryza sativa* (Piao et al., 2019), *Vitis labruscana* (Azuma et al., 2011), and *Brassica napus* (Li et al., 2020). In *Arabidopsis*, several MYB-related proteins were shown to be involved in the circadian clock (Nagel and Kay, 2012). Timing of CAB expression 1 (TOC1) is a key evening oscillator component of the transcriptional network that establishes the core mechanism of circadian rhythm in plants (Kolmos et al., 2008). REVEILLE8/LHY-CCA1-LIKE5 (RVE8/LCL5) was reported to be capable of binding to the promoter of *TOC1* and promoted clock-controlled *TOC1* gene expression to regulate circadian rhythms in *Arabidopsis* (Farinas and Mas, 2011; Rawat et al., 2011). As central components of the circadian oscillator, *CIRCADIAN CLOCK ASSOCIATED1* (*CCA1*) and *LATE ELONGATED HYPOCOTYL* (*LHY*) were indicated to function synergistically and redundantly in *Arabidopsis* circadian clock by negatively regulating *TOC1* expression (Makino et al., 2002; Lu et al., 2009). Some *MYB-related* genes, such as *EARLY-PHYTOCHROME-RESPONSIVE1* (*EPR1*) (Kuno et al., 2003), *KUODA 1* (*KUA1*) (Lu et al., 2014), *REVEILLE2* (*RVE2*) (Zhang et al., 2007), and *MYB HYPOCOYL ELONGATION-RELATED* (*MYBH*) (Kwon et al., 2013), were also revealed to participate in circadian clock regulation in *Arabidopsis*. *REVEILLE1 (RVE1)* is a critical node that integrates the circadian system and auxin signaling by regulating the expression of auxin biosynthetic gene *YUCCA8* (*YUC8*, also known as *CYTOKININ INDUCED ROOT CURLING2*) (Rawat et al., 2009). A previous study suggested that MYBL2 could interact with MYB-bHLH-WDR (MBW) complexes to regulate flavonoid biosynthesis, and *MYBD* promoted anthocyanin accumulation by regulating *MYBL2* expression in a circadian-dependent manner (Dubos et al., 2008; Nguyen and Lee, 2016). In addition to playing significant roles in circadian rhythm regulation, the MYB-related proteins also participate in the regulation of a broad range of developmental processes, such as seed germination (Zhao et al., 2019), trichome differentiation (Tominaga-Wada et al., 2017), leaf development and senescence (Piao et al., 2019), shoot apical meristem (SAM) formation (Lin et al., 2007), root development (Li et al., 2020), and flowering (Shibuta and Abe, 2017). A single-repeat MYB-related transcription factor TRIPTYCHON (TRY), was shown to be expressed in trichomes and mediated trichome patterning and position-dependent cell determination in root hair formation in *Arabidopsis* (Schellmann et al., 2002). *MYB-related c1/pl1* genes were revealed to act as regulators of anthocyanin synthesis in maize in response to different light qualities and cytokinin (Piazza et al., 2002; Pilu et al., 2003). Grape *VvmybA1-1, VvmybA1-2*, and *VvmybA2* were isolated from *V*. *labruscana*, and were indicated to manipulate the skin colors of grapes by regulating anthocyanin biosynthesis in grape berries (Azuma et al., 2008). The maize MYB-related protein-1 (ZmMRP-1), which is specifically expressed in the transfer cell layer of the endosperm, was reported to play a role in determining the differentiation of transfer cells by transcriptionally activating the expression of maize endosperm transfer cell-specific gene *BETL1*, and this process relied on the interaction between ZmMRP-1 and two C_2_H_2_ zinc finger proteins (ZmMRPI-1 and ZmMRPI-2) (Royo et al., 2009). Overexpression of the *MYB-related* gene, *OsMYB102* in rice delayed leaf senescence, and OsMYB102 was suggested to modulate the expression of various kinds of senescence-associated genes, including genes participating in abscisic acid (ABA) metabolism and signaling (Piao et al., 2019). *FE*, which encodes a phloem-specific MYB-related protein, was documented to positively regulate the transcription level of *FLOWERING LOCUS T* (*FT*) and *FLOWERING LOCUS T INTERACTING PROTEIN 1* (*FTIP1*), thereby promoting flowering in *Arabidopsis* (Abe et al., 2015). MYB-related *AtCDC5* was proven to be critical for G2 to M transition of the cell cycle and controls the function of SAM in *Arabidopsis* (Lin et al., 2007). A wheat *MYB-related* gene, *TaMYB72*, was indicated to promote flowering in rice *via* upregulation of florigen genes, *Hd3a* and *RFT1* (Zhang et al., 2016).

Notably, *MYB-related* genes were also demonstrated to play significant roles in improving abiotic stress tolerance in plants. A large number of *MYB-related* genes respond to diverse abiotic stresses and are crucial for plant resistance to adverse environmental conditions (Shi et al., 2015; Yong et al., 2019). A rice *MYB-related* gene, *OsMYB48-1* was reported to regulate drought and salt tolerance positively by regulating ABA biosynthesis (Xiong et al., 2014). Overexpression of *OsMYBR1* conferred improved drought tolerance and reduced ABA sensitivity to rice (Yin et al., 2017). Heterologous expression of a *Fraxinus velutina* gene, *FvMYB1*, in tobacco enhanced salt tolerance of the transgenic tobacco plants (Li et al., 2016). A recent study showed that RSM1 regulates seed germination and seedling development under ABA treatment and salt stress conditions by interacting with HY5/HYH (Yang et al., 2018). Overexpression of lily *LlMYB3* in *Arabidopsis* increased cold, salt, and drought tolerance of the transgenic plants (Yong et al., 2019). Sheepgrass LcMYB2 was suggested to positively regulate drought tolerance by promoting root growth and osmotic adjustment (Zhao et al., 2019). Moreover, overexpression of *GmMYB118* enhanced drought and salt tolerance in both transgenic *Arabidopsis* and soybean seedlings (Du et al., 2018). *MYB-related* genes were also revealed to be involved in cold tolerance. For example, the GARP-like transcription factor, VaAQUILO (VaAQ) was reported to dramatically improve cold tolerance by increasing the content of raffinose family oligosaccharides (RFOs) and osmoprotectants in transgenic *Arabidopsis* and in Amur grape calli (Sun et al., 2018).

Rapeseed is the third largest oil crop globally, and is a vital agricultural resource (Liao et al., 2019). Previous transcriptomic analysis revealed that *MYB-related* genes might be involved in the growth and development of *B. napus* (Hong et al., 2017; Li et al., 2020). However, systematic analysis of MYB-related proteins and the research demonstrating the roles of *MYB-related* genes in *B. napus* are still lacking. In the present study, various *in silico* approaches were applied to identify *BnMYB-related* genes, and 251 genes encoding BnMYB-related proteins were identified in *B. napus*. The phylogenetic relationships, gene structures, *cis*-elements, temporospatial expression patterns, and expression profiles of *BnMYB-related* genes under various stress conditions were studied comprehensively. Considering that little is known about the roles of *MYB-related* genes in *B. napus*, the results of this study will provide a useful reference for further understanding of the functions of *BnMYB-related* genes in response to abiotic stresses and in the regulation of developmental processes in *B. napus*.

## Materials and Methods

### Identification of *MYB-Related* Genes in the *B. napus* Genome

The genome and protein sequences of *B. napus* (Darmor-bzh) were obtained from the Genoscope database (http://www.genoscope.cns.fr/brassicanapus/). Conserved DNA binding domains of known MYB-related proteins were employed to search against the *B. napus* database (https://www.arabidopsis.org/) using the BLASTP program with an *E* value of less than 1e^−10^ to identify BnMYB-related proteins. Meanwhile, “MYB conserved domain” and “Myb_DNA-binding” were used as keywords to query the Pfam database (https://pfam.xfam.org/) (El-Gebali et al., 2019), and the corresponding Hidden Markov Model (HMM) profile (PF00249) was downloaded and employed to identify MYB-related proteins from *B. napus* using the HMMER tool (https://www.ebi.ac.uk/Tools/hmmer/). Moreover, NCBI-Conserved Domain Data (CDD) search (https://www.ncbi.nlm.nih.gov/Structure/cdd/wrpsb.cgi) and InterproScan (http://www.ebi.ac.uk/interpro/search/sequence-search) were used to analyze and classify the BnMYB-related protein sequences. Then, the non-redundant sequences were compared with the MYB-related family in the plant transcription factor (TF) database version 4.0 (PlantTFDB 4.0, http://planttfdb.cbi.pku.edu.cn/) (Jin et al., 2017). Conserved domains of the retrieved putative MYB-related proteins were verified manually. Detailed information for the *BnMYB-related* genes is listed in Supplementary Table 1. ExPASy (https://web.expasy.org/protparam/) was used to calculate the number of amino acids, molecular weights (MW), and theoretical isoelectric points (pI) of BnMYB-related proteins.

### Multiple Sequence Alignments and Phylogenetic Analysis

Multiple alignments of full-length BnMYB-related protein sequences were conducted using ClustalX (version 1.83) (Thompson et al., 1997). A phylogenetic tree was constructed using MEGA 7.0 software (https://www.megasoftware.net/) with the neighbor-joining (NJ) method, and bootstrap test was performed with 1,000 iterations, as described previously (Wang et al., 2017).

### Gene Structure and Motif Analysis

Gene structure data were obtained from the *B. napus* genome annotation file on the Genoscope database. Exon-intron organizations of *BnMYB-related* genes were shown by the gene structure display server program (GSDS2.0, http://gsds.cbi.pku.edu.cn/) based on the alignment results of the complementary DNA (cDNA) sequences with the corresponding genomic DNA sequences of each gene. The MEME (http://meme-suite.org/tools/meme) program was used to identify the conserved motifs in BnMYB-related protein sequences. The motif distribution type was set as zero or one occurrence per sequence. The number of motifs was set as 20, and the motif width was between 6 and 50 amino acids. These data were integrated and visualized using TBtools (Chen et al., 2020).

### Gene Location and Duplication

Location information of *BnMYB-related* genes was obtained from the *B. napus* genome annotation file on the Genoscope database. *BnMYB-related* genes were mapped to the *B. napus* genome using the MG2C (http://mg2c.iask.in/mg2c_v2.0/) online tools. Moreover, the whole-genome protein sequences from rapeseed were blasted against *B. napus, B. rape, B. oleracea*, and *A. thaliana* databases with an *E* value of less than 1e^−10^. MCScanX was used to detect the collinear blocks with default parameter settings (Wang et al., 2012). *Ka*/*Ks* ratios were calculated by ParaAT2.0 and KaKs_Calculator 2.0 programs (Wang et al., 2010; Zhang et al., 2012). The collinear blocks of *BnMYB-related* genes were obtained. In addition, the homolog pairs of *MYB-related* genes in eight rapeseed accessions of three ecotypes (winter-type, semi-winter-type, and spring-type) including ZS11, Gangan, No2127, Quinta, Shengli, Tapidor, Westar, and Zheyou7 were captured from the BnPIR database (*Brassica napus* pan-genome information resource, http://cbi.hzau.edu.cn/bnapus/) (Song et al., 2020). The Upset plot and colinearity between them were visualized using TBtools (Chen et al., 2020). The putative duplicated genes were linked with arcs in the relevant figure.

### Regulatory *Cis*-Element Analysis

A 2-kb upstream sequence of coding regions of each *BnMYB-related* gene was selected as the promoter region for *cis*-element analysis. The Samtools program (http://www.htslib.org/) was used to obtain the promoter sequences from the *B. napus* genome, which were searched against the plant *cis*-acting regulatory DNA elements database (PlantCARE, http://bioinformatics.psb.ugent.be/webtools/plantcare/html/) to identify the putative *cis*-acting regulatory elements (Lescot et al., 2002).

### Plant Materials, Growth Conditions, and Treatments

To examine the expression patterns of *BnMYB-related* genes in various *B. napus* tissues and organs, and under a variety of abiotic stress conditions, rapeseed seeds were germinated on wet filter papers. Germinated rapeseed seedlings were then transplanted to the field or a growth chamber with a 16 h light/8 h dark photocycle. For temporospatial expression analysis, the rapeseed plants were grown under field conditions over the whole growth period. Cotyledons, roots, stems, young leaves, SAM, mature leaves, flowers, ovaries, silique walls, and seeds at different stages [14 days, 24 days, 34 days, and 50 days after pollination (DAP)] were sampled. In order to establish the expression profile under various stress conditions, 4-week-old *B. napus* seedlings cultured in nutrient solutions in a glasshouse were subjected to various treatments, including drought, heat, salt, cold stress, and ABA treatment. For drought treatment, seedlings were taken out from the nutrient solution and dehydrated, and the leaves were sampled at 0.5 h after treatment. For salt treatment, seedlings were transferred to nutrient solutions containing 200 mM NaCl and sampled at 6 h after treatment. For cold and heat treatment, rapeseed seedlings were transferred to growth chambers at 4 and 42°C, and sampled at 6 and 1 h after treatment, respectively. For ABA treatment, seedlings were sprayed with 100 μM ABA and sampled at 6 h after treatment. Three biological replicates were performed for each experiment with five plants for each biological replicate.

### RNA Isolation and Quantitative Real-Time Reverse Transcription PCR (qRT-PCR) Analysis

Plant tissues/organs were frozen in liquid nitrogen and ground into a fine powder using a mortar. Total RNA was extracted using an RNAprep pure plant kit (TIANGEN Biotech, Beijing, China) according to the instructions of the manufacturer. Then, 4 μg of total RNA of each sample was reverse transcribed using the HiScript® II 1st Strand cDNA Synthesis Kit (+gDNA wiper) (Vazyme, Nanjing, China) to produce cDNA. For qPCR analysis, gene-specific primers were designed using PrimerExpress v3.0 (Applied Biosystems, Foster City, CA, USA). The qPCR assay was carried out using PowerUp^TM^ SYBR^TM^ Green Master Mix (Applied Biosystems, Waltham, MA, United States) according to the instructions of the manufacturer on StepOnePlus^TM^ Real-time PCR instrument (Applied Biosystems, Waltham, MA, United States). The *BnActin* gene was used as an internal control. Three technical replicates were conducted for each sample. The reaction procedure was as follows: 50°C for 2 min and 95°C for 5 min; followed by 40 cycles of 95°C for 15 s, 58°C for 15 s, and 72°C for 30 s. Relative expression of *BnMYB-related* genes was calculated as described previously (Livak and Schmittgen, 2001). The primers used for PCR are listed in Supplementary Table 1.

### Gene Cloning and Vector Construction

The full-length coding region of *BnMRD107* (without its stop codon) was amplified and inserted into pMDC83 vector under the control of *CaMV35S* promoter to express a BnMRD107-GFP fusion protein, and the construct was designated as *BnMRD107*-OE. The primers used in this experiment are listed in Supplementary Table 1.

### Generation of Transgenic *B. napus* Plants

Genetic transformation of *B. napus* was based on the *Agrobacterium*-mediated hypocotyl transformation method described previously (Dai et al., 2020), and a pure line semi-winter rapeseed J9712, was used as the transformation recipient. The regenerated *B. napus* plants were screened on a medium containing 25 mg/L hygromycin B.

### Phenotyping of *B. napus* Plants for Osmotic and Salt Stress Tolerance at the Cotyledon Stage

The T_2_ generation of homozygous transgenic *BnMRD107*-OE lines in rapeseed “J9712” background was chosen for further analysis. For rapid identification of the performance of transgenic materials and control plants under various stress conditions, stress treatments on the culture medium at the cotyledon stage were carried out. Untransformed J9712 was used as the control. For osmotic and salt tolerance assays at the cotyledon stage, selfed seeds of *BnMRD107*-OE lines and J9712 were surface-sterilized with 70% ethyl alcohol and 10% sodium hypochlorite, and then germinated on plates comprising 1/2 Murashige and Skoog (MS) medium. The seeds were incubated at 22°C, 16 h light/20°C, 8 h dark cycle until germinated. Germinated seeds with the same growth vigor were then transferred to 1/2 MS medium, and 1/2 MS medium containing 150 mM mannitol (for osmotic stress) and 150 mM NaCl (for salt stress), respectively. The rapeseed materials were cultured in a 22°C, 16 h light/20°C, 8 h dark cycle for 5 days. Seedlings were photographed, and their hypocotyl length, root length, and fresh weight were measured. Three independent biological replicates were performed for each experiment with five seedlings.

### 3,3′-Diaminobenzidine (DAB) Staining

3,3′-diaminobenzidine (DAB) DAB staining of cotyledons of *BnMRD107*-OE and J9712 seedlings under normal, osmotic, and salt stress conditions was performed following a method described previously (Liu Y. et al., 2020).

## Results

### Identification of BnMYB-Related Proteins

Genome sequence of *B. napus* was downloaded from the GENOSCOPE database. BLASTP searching was applied to identify BnMYB-related proteins using the DNA-binding domain of known MYB-related proteins against the *B. napus* database. Putative MYB-related proteins in *B. napus* were also retrieved based on HMMER model using the PF00249 HMM profile. NCBI-CDD searching and InterproScan were used to test the dependability of the results. A total of 251 *BnMYB-related* genes were identified, with 120 and 131 genes from the AA- and CC-subgenomes, respectively (Supplementary Table 2). Validated *BnMYB-related* genes were named as *BnMRD1*-*BnMRD251* based on their sequential locations on the *B. napus* chromosomes to keep the nomenclature consistent.

### Molecular Characteristics of BnMYB-Related Proteins and Chromosomal Localization of *BnMYB-Related* Genes

Molecular characteristics, including the protein length, MW, and isoelectric point (pI) of BnMYB-related proteins were analyzed (Supplementary Table 2). The protein lengths of BnMYB-related proteins ranged from 51 (BnMRD54) to 1,885 amino acids (BnMRD27). The MWs of BnMYB-related proteins ranged from 5.67 (BnMRD54) to 208.31 kDa (BnMRD27). The pIs of 171 BnMYB-related proteins (68.92%) were >7, suggesting that these proteins were basic proteins, and the remaining 31.08% of the proteins were acidic proteins. Locations of all *BnMYB-related* genes on the *B. napus* chromosomes are shown in Supplementary Figure 1. Total of 251 *BnMYB-related* genes were distributed in 19 chromosomes (i.e., A01–A10, and C01–C09), whereas 34 members (e.g., *BnMRD5, BnMRD111, BnMRD159*, and *BnMRD183*) were not shown on the *B. napus* chromosomes because they were located on unanchored scaffolds, which were unable to map onto the specific chromosomes. As shown in Supplementary Figure 1, *BnMYB-related* genes were unevenly distributed on 19 chromosomes. The number of *MYB-related* genes distributed on C03 was the highest, whereas that distributed on A04 was the lowest.

### Gene Structures, Motif Identification, and Phylogenetic Analysis

**Figure 1:**
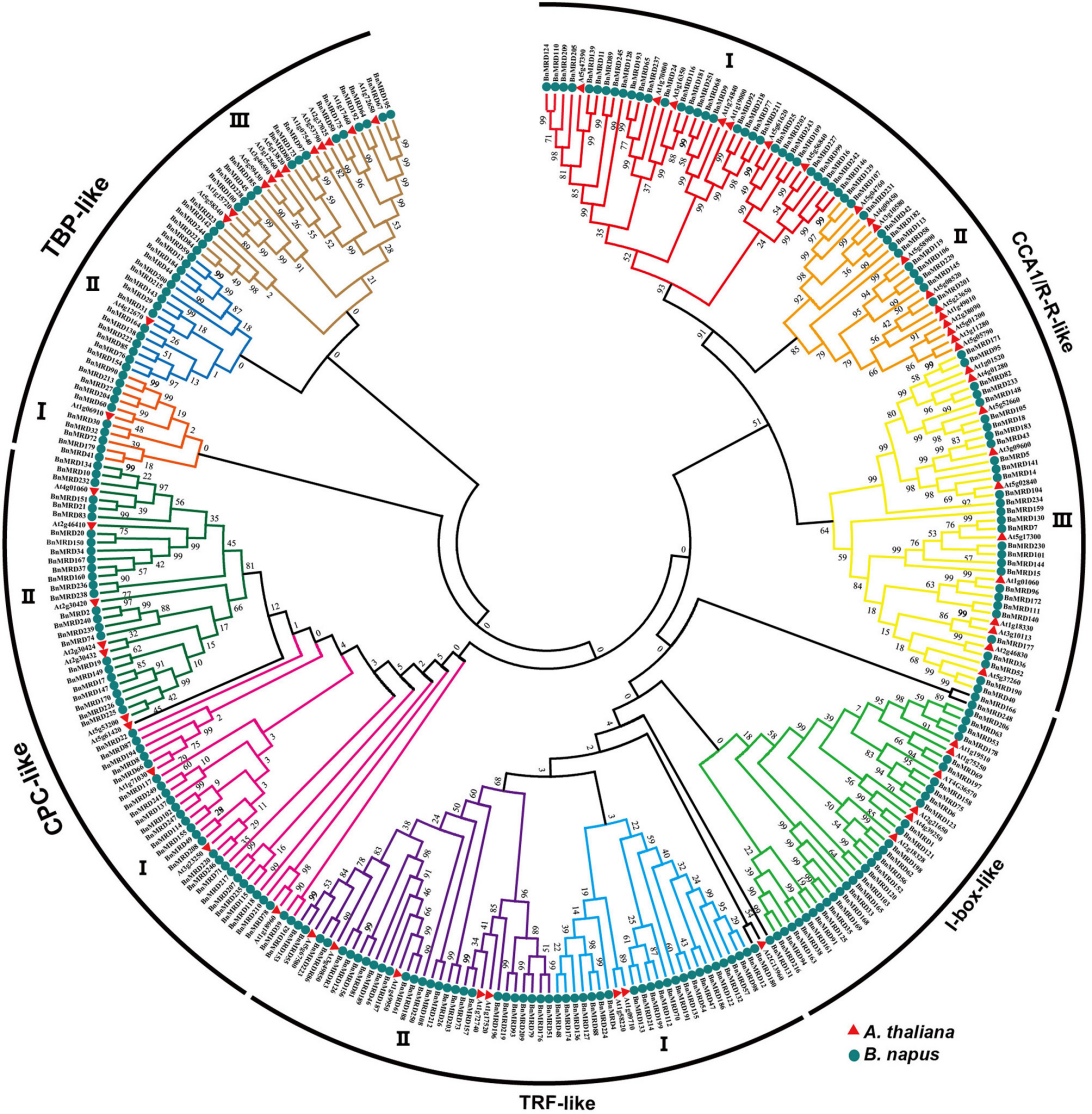
Phylogenetic analysis of MYB-related proteins using MEGA 7.0 with the neighbor-joining (NJ) method. Numbers at the nodes represent the reliability percentage of the bootstrap values based on 1,000 replications. Red triangles and green circles represent the MYB-related proteins in *Arabidopsis thaliana* (*A. thaliana*) and *Brassica napus* (*B. napus*), respectively.

A neighbor-joining (NJ) phylogenetic tree of MYB-related family was constructed to investigate the evolutionary relationship of MYB-related proteins using the full-length sequences of MYB-related proteins. All the 251 MYB-related proteins were classified into five distinct clades, named as CCA1/R-R-like, I-box-like, TRF-like, CPC-like, and TBP-like, respectively (Figure 1). CCA1/R-R-like clade was the largest clade. To determine the structural conservation and diversification of MYB-related family, the exon-intron structures of each individual *BnMYB-related* gene were characterized, and an online MEME analysis tool was applied to identify the conserved motifs of BnMYB-related proteins.

**Figure 2:**
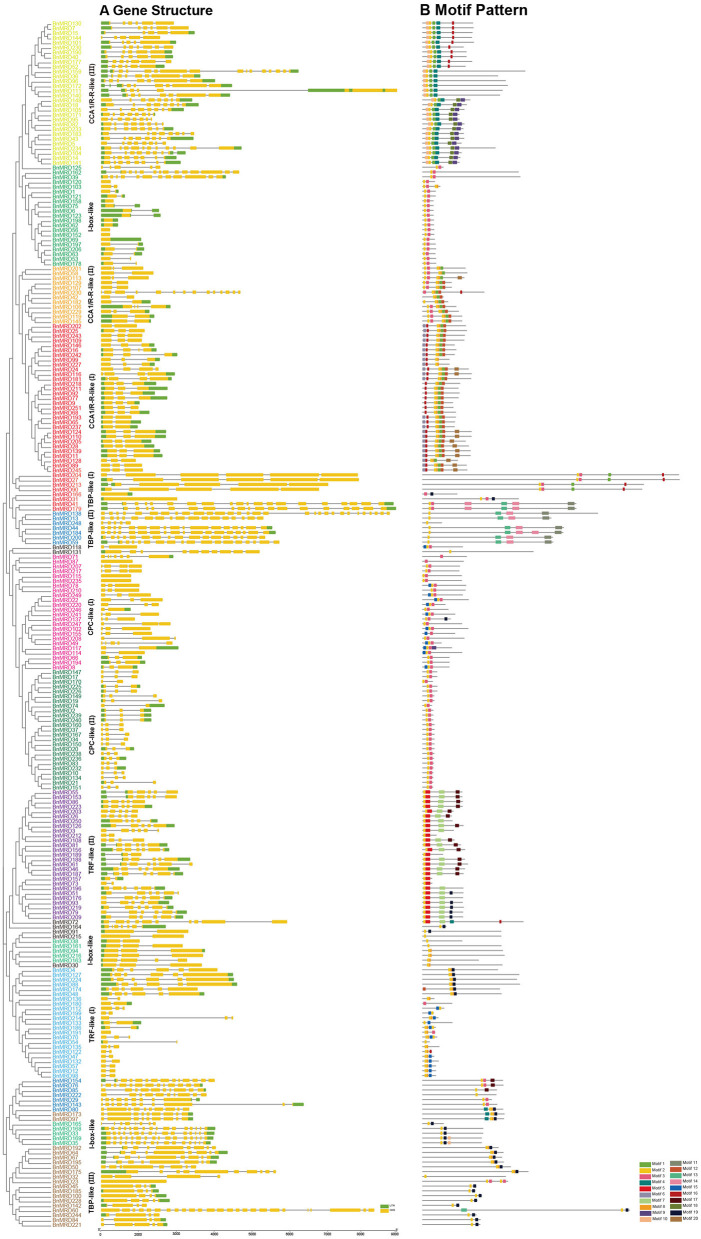
Gene structures and motif compositions of MYB-related proteins in *B. napus*. **(A)** Exon-intron structures of BnMYB-related proteins. Green boxes indicate 5′- and 3′-untranslated regions (UTR), respectively. Yellow boxes indicate exons, and black lines indicate introns. **(B)** Distribution of conserved motifs in BnMYB-related proteins. Motifs 1–20 identified by MEME analysis are displayed in different colored boxes.

The number of introns of *MYB-related* genes varied dramatically in *B. napus* (Figure 2A). BnMYB-related proteins were subdivided into smaller subgroups (I, II, III, and IV) according to the characteristics of motif composition and the exon-intron structures of their encoding genes. The numbers of exons ranged from 1 to 30 (Figure 2A). Nine genes of I-box-like clade had no introns. Most of the members of CCA1/R-R-like (I, II) group had 2–3 introns, while the members of CCA1/R-R-like (III) group had 8–15 introns. The majority of members of CPC-like clade had 2–3 introns. TBP-like group genes had the most complex exon-intron structure, with up to 24 introns. In general, there was no distinct regularity in the intron distribution pattern among the *BnMYB-related* genes; however, members belonging to the same clade shared identical or similar intron phases and motif compositions (Figure 2).

Sequence alignment revealed that the conserved core sequence of MYB domains in MYB-related proteins belonging to different clades was remarkably divergent, whereas the tryptophan (W) residues were found to be evenly distributed in the conserved MYB repeat regions in most subgroups of MYB-related proteins. There were three W residues in MYB-domains of TRF-like (II) and CPC-like (II) members. Substitution or deletion at the first or the third W residue in MYB domain was observed in more than 20% of BnMYB-related proteins. For instance, members of CCA1/R-R-like subgroup had a conserved sequence of SHAQKY(N)F or SHAQK, in which the third W residue was substituted by Alanine (A). The third W residue was substituted by tyrosine (Y) in I-box-like clade proteins. The amino acid sequences of MYB domain were found to vary significantly in different subgroups in TBP-like clade members. However, a conserved sequence of DLKDKW(R/K) (N/T) was found in TBP-like (III) subgroup proteins. The conserved and subgroup-specific core sequences of MYB domains indicated that members in the same subgroup had a common ancestor during evolution (Figure 2). As shown in Figure 2B, 20 motifs were recognized in BnMYB-related proteins, with lengths of 11 to 50 amino acids. Motif 2 was the most conserved motif, being present in all the family members, which made up the core sequence of the single MYB domain. Motif 1, with the conserved SHAQKY sequence, was a distinct motif that existed in CCA1/R-R-like clade. CCA1/R-R-like (I) subgroup members generally harbored motif 1, motif 2, motif 6, motif 8, motif 12, and motif 16. Interestingly, N-termini of the proteins in this subgroup were relatively conserved. Motif 1, motif 2, motif 3, motif 8, and motif 12 were identified in CCA1/R-R-like (II) subgroup. Besides, motif 4 (QKSGTNIHIPPPRPKRKPAHPYPRKAGKN) and motif 10 (SGEDLAKKVRKPYTITKSRER) were considered as the basis to distinguish CCA1/R-R-like (III) members from members of the other two subgroups in CCA1/R-R-like clade. Proteins of I-box-like clade had the simplest motif composition with only two motifs (motif 2 and motif 3). TRF-like clade was divided into two subgroups. Motif 15 was found in 11 TRF-like (I) members and motif 19 was found in five TRF-like (II) members. Motif 3, motif 5, and motif 7 were the most common motifs in TRF-like (II) proteins. Motif 19 and motif 17 were also found in several members of TRF-like (II) subgroup. CPC-like clade also had two subgroups. CPC-like (I) proteins exhibited a similar motif composition (motif 2 and motif 3) to those of I-box-like proteins; however, the locations of conserved motifs in CPC-like (I) and I-box-like proteins were different. TBP-like clade proteins were basically classified into three subgroups. Multiple motifs were detected in each TBP-like subgroup, and the distribution of the motifs was relatively scattered in the proteins.

**Figure 3:**
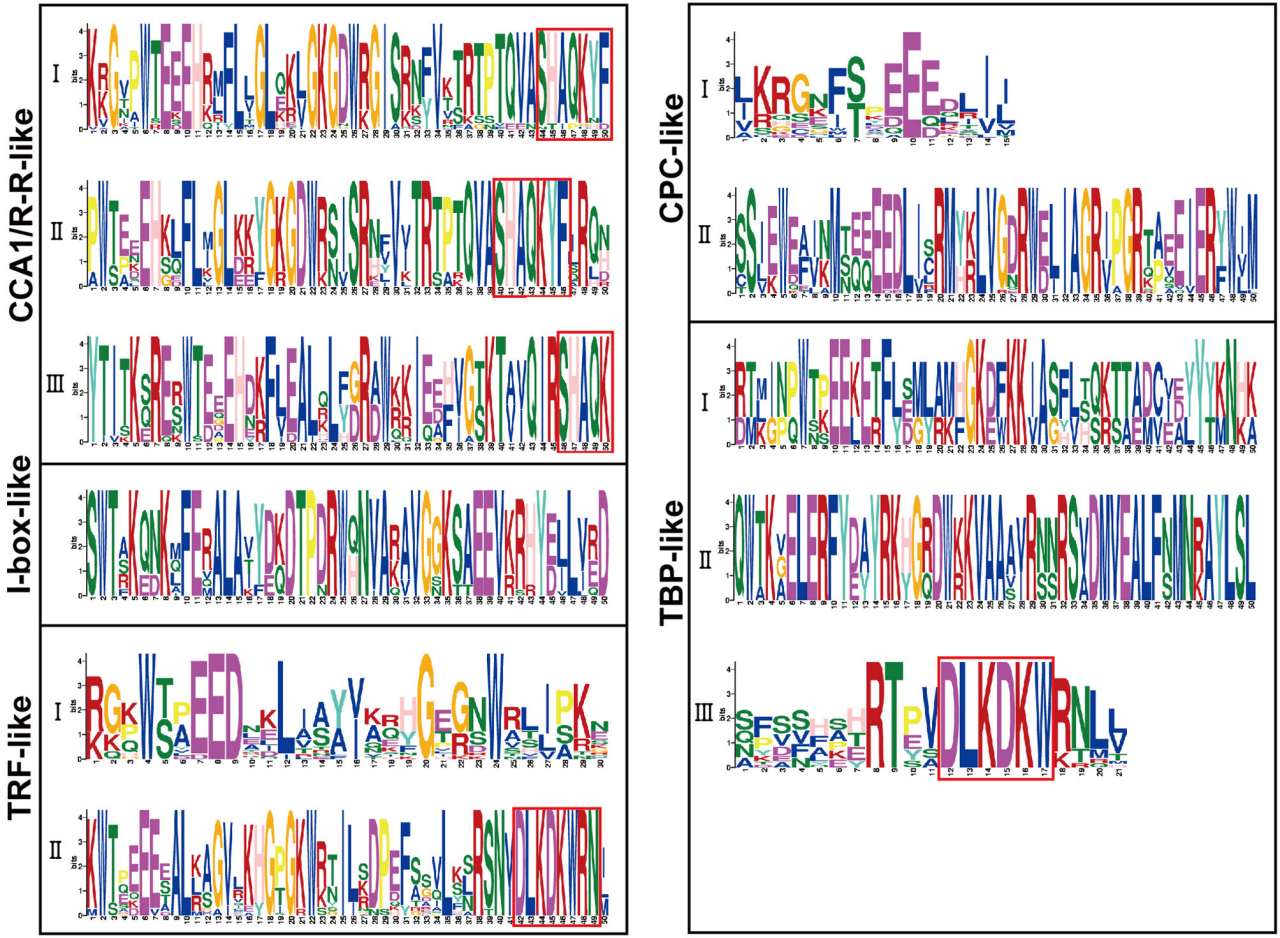
Amino acid sequence logos of the MYB domains in BnMYB-related proteins. Bit score indicates the information content for each position in the sequence. The conserved Trp (W) residues distributed in the amino acid sequence of MYB domains, and red boxes indicate the conserved motifs. The corresponding clades based on the phylogenetic tree (Figure 2) are indicated on the left for reference.

CCA1/R-R-like clade, with 73 members, was the largest of the five clades, and this clade could be further divided into three subgroups according to the exon-intron structure and motif characteristics. Among the 73 CCA1/R-R-like proteins, 31, 12, and 30 members belonged to group I, group II, and group III, respectively. All 73 members shared a highly conserved motif, SHAQKY(N)F or SHAQK, which is located in the third helix of MYB domain (Figure 3). Most of the MYB-related family members had a single MYB domain. However, CCA1/R-R-like (II) proteins in rapeseed contained two separate MYB domains, which are located in the N-terminus and the middle of the protein, respectively. This contrasted with the situation in 2R-MYB proteins, in which the two MYB domains are both distributed in the N-terminus of the proteins. TBP-like clade was the second largest, comprising 52 members. TBP-like clade had four subgroups with different exon-intron patterns and other conserved features. Ten, seven, 20, and 15 members fell into group I, group II, group III, and IV, respectively. Members of TBP-like (III) shared a consensus motif, DLKDKW(R/K) (N/T). Members of TBP-like clade had complicated exon-intron patterns (Figure 2). Both TRF-like and CPC-like clades comprised two subgroups. Most members of TBP-like (III) subgroup had two or three exons, while the majority of TBP-like (I) subgroup members had five to six exons (Figure 2). All of the individual *MYB-related* genes could be assigned into a clade based on phylogenetic analysis, except for *BnMRD131*. As shown in Figure 2, *BnMRD131* had a complex exon-intron organization and a distinct motif composition, which supported the hypothesis that *BnMRD131* was an orphan in BnMYB-related family. On the whole, most of the adjacent members in the phylogenetic tree exhibited similar exon-intron organizations and motif compositions.

### Synteny Analysis of MYB-Related Genes

The BLASTP and MCScanX programs were used to identify the homologous genes in BnMYB-related family based on synteny analysis. A total of 179 pairs of MYB-related paralogs were found in the *B. napus* genome (Supplementary Figure 2, Supplementary Table 3). Three tandemly duplicated genes, 194 pairs of whole genome duplication (WGD) or segmentally duplicated genes, 51 dispersedly duplicated gene pairs, and three proximal gene pairs were detected among BnMYB-related family (Supplementary Table 4). These results indicated that substantial numbers of *BnMYB-related* genes were generated by WGD or segmental duplication events, which played essential roles in the process of BnMYB-related family evolution.

**Figure 4:**
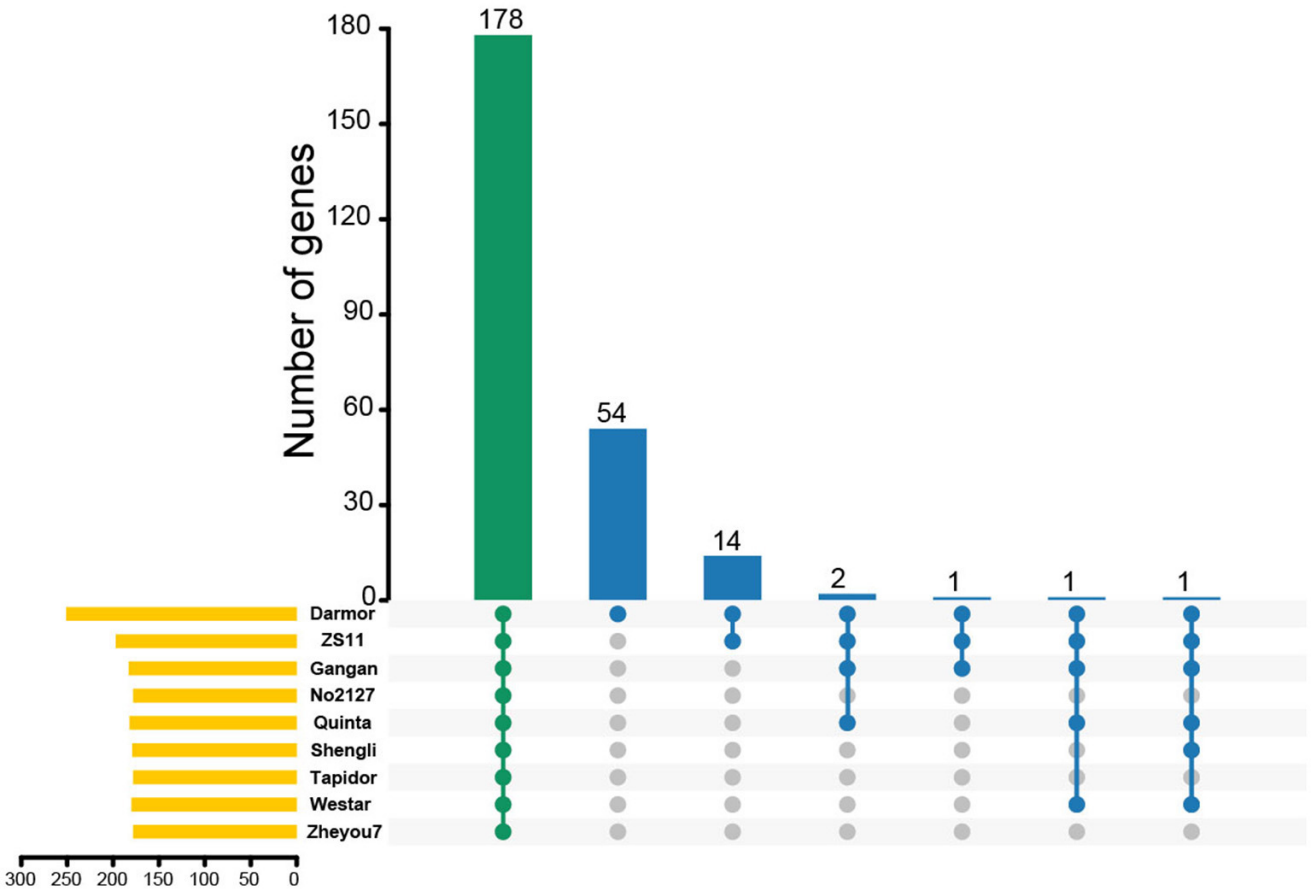
The Upset plot shows the distributions of homologous *MYB-related* genes in different rapeseed accessions. The bar chart above represents the number of common homologous genes shared among different rapeseed accessions. The left bar chart at the bottom represents the number of homologous genes in each accession. The right dotted line at the bottom shows the number of accessions contained in the group.

To further demonstrate the expansion and clustering of MYB-related family members, chromosomal syntenic genes of *BnMYB-related* genes in *Brassicaceae*, including *Arabidopsis, B. oleracea*, and *B. rapa* were analyzed. About 45.42% (114/251) of *BnMYB-related* genes had a syntenic relationship with *MYB-related* genes in the other three *Brassicaceae* species. 64, 76, and 84 orthologous genes located in syntenic blocks were identified from *Arabidopsis, B. oleracea*, and *B. rapa*, respectively (Supplementary Figure 2, Supplementary Tables 5–7), indicating that the expansion of BnMYB-related family was accompanied by gene loss. However, 91 *BnMYB-related* genes might be newly generated, which suggested that WGD played an essential role in the expansion of *BnMYB-related* gene family. Moreover, the homolog pairs of *MYB-related* genes from eight rapeseed accessions were identified. The results suggested that a total of 178 *MYB-related* genes were conserved in all eight rapeseed accessions, while 54 *MYB-related* genes were specific to Darmor-*bzh* cultivar. Besides the above-mentioned 178 common members in eight accessions, 14 genes were shared between Darmor-*bzh* and ZS11, and 1–2 genes were shared in other accessions (Figure 4). The corresponding relationships of MYB-related homologs among the eight rapeseed accessions are listed in Supplementary Table 8. The homologs of 78.09% (196/251), 72.91% (183/251), 70.92% (178/251), 72.51% (182/251), 71.31% (179/251), 70.92% (178/251), 71.71% (180/251), and 70.92% (178/251) of *MYB-related* genes in Darmor-*bzh* were identified by alignment in ZS11, Gangan, No2127, Quinta, Shengli, Tapidor, Westar, and Zheyou7, respectively. Overall, these results demonstrated that WGD events were the major forces driving the expansion of *MYB-related* genes in *B. napus* and the MYB-related family is largely conserved in different rapeseed accessions.

To gain a deeper insight into the evolutionary selective constraints on duplicated *BnMYB-related* genes, ParaAT2.0 (https://bigd.big.ac.cn/tools/paraat) was used to calculate non-synonymous (*Ka*) and synonymous (*Ks*) values, and the *Ka*/*Ks* substitution ratios. The *Ka*/*Ks* ratios were lower than 1 in all identified BnMYB-related segmental duplications, and the divergence time ranged from 0.515 (BnMRD215 and BnMRD32) to 0.0028 (BnMRD16 and BnMRD146) million years ago (Supplementary Table 3). This result provided evidence of strong purifying selection pressure on these duplicate genes in rapeseed. The *Ka*/*Ks* ratios were also calculated to evaluate the selection pressure among duplicated *MYB-related* gene pairs of *A*. *thaliana* vs. *B. napus, B*. *oleracea* vs. *B. napus*, and *B*. *rapa* vs. *B. napus* (Supplementary Tables 5–7). The *Ka*/*Ks* values from all the identified gene pairs were less than 1, indicating that MYB-related family in *B. napus* and *A*. *thaliana*, as well as in *B. napus* and its diploid progenitors, experienced purifying selection during family expansion.

### *Cis*-Element Analysis of the Promoter Regions of *BnMYB-Related* Genes

**Figure 5:**
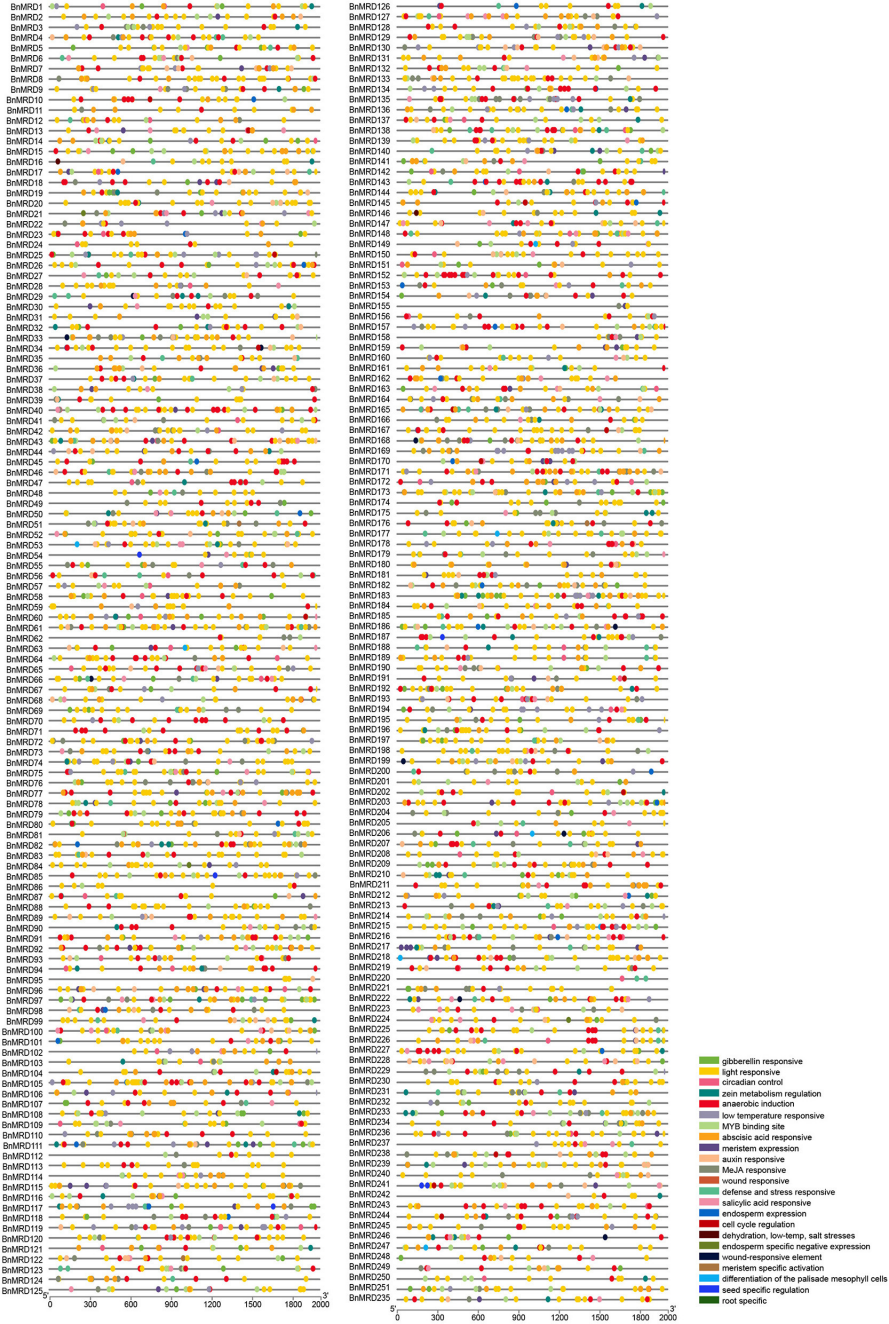
Regulatory *cis*-elements on the promoter regions of *BnMYB-related* genes. Locations of different *cis*-acting elements are arranged on the lines representing the 2 kb upstream region of TSS in the promoters of each *BnMYB-related* gene. Different colored ovals represent different types of *cis*-elements.

A 2-kb genomic sequence upstream of the transcription start site (TSS) of each *BnMYB-related* gene was used to probe the regulatory *cis*-acting elements enriched in *BnMYB-related* genes using the plant CARE database. As shown in Figure 5 and Supplementary Table 9, 23 types of *cis*-acting elements were identified in the promoters of *BnMYB-related* genes. The existence of abundant *cis*-acting elements in the promoter regions of *BnMYB-related* genes might have implications for their transcriptional regulation and biological functions. The identified *cis*-acting elements were mainly divided into three types. Eleven elements including light responsive motif (GT1-motif), circadian control, zein metabolism regulation, meristem expression (CAT-box), endosperm expression (GCN4-motif), cell cycle regulation, endosperm specific negative expression (AACA-motif), meristem-specific activation (NON-box), differentiation of the palisade mesophyll cells, seed-specific regulation (RY-element), and root-specific *cis*-elements, were associated with plant growth and development. Light responsive elements appeared to be the most common *cis*-acting elements in *BnMYB-related* gene promoters, suggesting that *BnMYB-related* genes might play a vital role in the response of *B. napus* to variable light conditions. Several *cis*-elements, such as meristem-specific activation, seed-specific regulation, and root-specific elements, were considered to be associated with temporospatial-specific expression of the *BnMYB-related* genes. Five elements including gibberellin-responsive (GARE), ABA responsive (ABRE), auxin responsive (TGA), methyl jasmonate (MeJA)-responsive (TGACG-motif), and salicylic acid responsive elements (TCA) were responsible for phytohormone responses. Five elements including anaerobic induction (ARE), wound responsive (WUN-motif), defense and stress responsive (TC-rich repeats), low-temperature responsive (LTR), and dehydration, low-temperature, salt stress-responsive were involved in stress responses, implying that the *BnMYB-related* genes containing these *cis*-elements in their promoter regions might participate in the adaptation of *B*. *napus* to everchanging environmental conditions. Interestingly, an anaerobic induction element appeared as tandem repeats in some of the *BnMYB-related* gene promoters (*BnMRD10, BnMRD40, BnMRD45, BnMRD47, BnMRD70, BnMRD71, BnMRD134, BnMRD118, BnMRD207, BnMRD225, BnMRD226*, and *BnMRD227*), hinting that the regulatory proteins which bind to these regions might have special spatial structures. It is noteworthy that an MYB binding site was found in the promoter region of most *BnMYB-related* genes, revealing that the transcriptional abundance of *BnMYB-related* genes was largely modulated by MYB transcription factors.

### Temporospatial Expression Profiles of *BnMYB-Related* Genes

**Figure 6:**
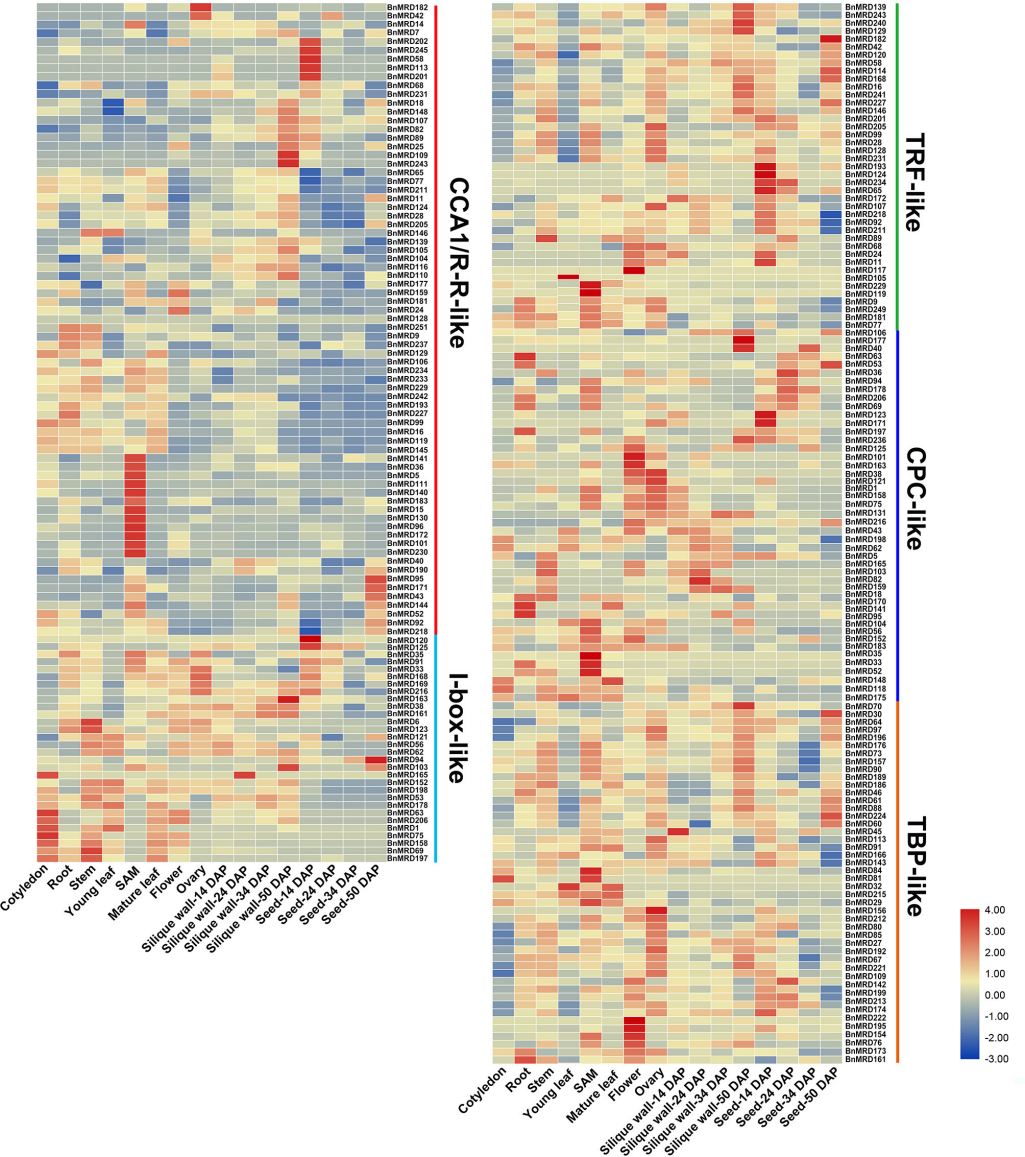
Temporospatial expression patterns of *BnMYB-related* genes. Fragments per kilobase of transcript per million mapped reads (FPKM) values of *BnMYBR* genes are log_2_ transformed and are used to represent the gene expression levels. The horizontal axis represents 16 tissues/organs of different developmental stages in rapeseed. The color scale represents gene expression levels with high transcript abundance (red) or low transcript abundance (blue).

To dissect the expression characteristics of *BnMYB-related* genes, their transcript patterns in tissues and organs of the main developmental stages (cotyledon, root, stem, leaf, SAM, overleaf, flower, ovary, silique wall at 14, 24, 34, and 50 DAP, and seed at 14, 24, 34, 50 DAP) of *B. napus* were examined based on transcriptome data, and a visualization heatmap was generated (Figure 6, Supplementary Table 10). The results showed that *BnMYB-related* genes exhibited distinct expression patterns among the tissues and organs detected in this study, revealing their diverse functions during *B. napus* development. Among the *BnMYB-related* genes, 237 genes showed detectable expression in at least one tissue or organ. The remaining 14 *BnMYB-related* genes were not expressed in tested tissues or organs, 10 of which belonged to TRF-like clade, and four genes belonged to CPC-like clade. These genes might be non-functional, or else they have a highly specific expression pattern. Genes with undetectable expression are not shown in Figure 6. Transcripts of all CCA1/R-R-like genes were detectable in most tested tissues and organs, although their transcript abundance was relatively lower than that of genes from the other clades. It is important to note that 12 genes in CCA1/R-R-like clade (including *BnMRD36, BnMRD96, BnMRD101*, and *BnMRD111*) were preferentially expressed in SAM, suggesting that they might participate in SAM development. Ten genes including *BnMRD95, BnMRD109*, and *BnMRD202* showed high and specific expression in seeds. *BnMRD182* and *BnMRD42* were predominantly expressed in flowers. Approximately half of the I-box-like clade genes were predominantly expressed in vegetative tissues and organs (especially cotyledon) at the seedling stage, indicating that they probably participate in vegetative growth. TRF-like genes displayed a diverse expression pattern in the samples tested. The majority of TRF-like genes showed higher expression in the reproductive tissues and organs than in the vegetative tissues and organs and had relatively low transcript abundance in cotyledons and leaves. Many genes of CPC-like clade had high expression in SAM, flowers, and ovaries. Transcripts of TBP-like clade genes were detected in almost all tested samples, and half of them showed a relatively high expression in flowers and ovaries. These findings supported the view that *BnMYB-related* genes might play fundamental roles in the entire developmental process in rapeseed.

### Expression Profiles of *BnMYB-Related* Genes Under Abiotic Stress Conditions

**Figure 7:**
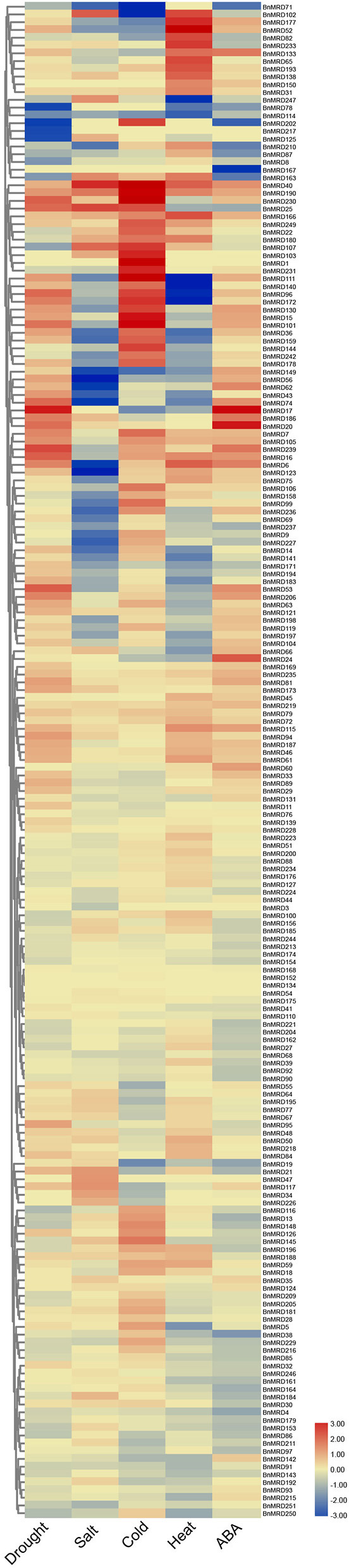
*BnMYB-related* genes in response to four abiotic stress conditions (drought, salt, cold, and heat) and ABA treatment. Ratios of FPKM values under stress conditions to FPKM values under normal conditions are log_2_ transformed and used to represent the gene expression levels.

Based upon the *cis*-element analysis, multiple abiotic stress response-associated elements were found in the promoter regions of *BnMYB-related* genes. To better understand the potential roles of *BnMYB-related* genes in responses to various abiotic stresses in *B. napus*, the expression patterns of individual members under drought, salt, cold, heat stress, and ABA treatment were investigated based on transcriptome data previously created by our lab (NCBI SRA BioProject ID: PRJNA687395 and PRJNA721476, Supplementary Table 11). A total of 195 *MYB-related* genes, more than half of all family members, displayed a stress-responsive expression pattern (Figure 7). The results showed that *BnMYB-related* genes had different expression patterns under four abiotic stresses and ABA treatment. Among them, 110 and 74 genes, 82 and 101 genes, 113 and 89 genes, 74 and 91 genes, were upregulated and downregulated by drought, salt, heat, and cold stress, respectively. The expression levels of 89 genes were elevated, whereas the expression levels of 96 genes were inhibited by ABA treatment. Further analysis showed that all four abiotic stresses and ABA treatment induced the expression of *BnMRD40, BnMRD190*, and *BnMRD166*. *BnMRD17, BnMRD20*, and *BnMRD186* were upregulated, whereas *BnMRD87, BnMRD78, BnMRD163*, and *BnMRD202* were downregulated by both drought stress and ABA treatment. Among *MYB-related* genes, 45.64% (89/195) (including *BnMRD13, BnMRD116*, and *BnMRD126*) showed increased expression under cold stress. Several BnMYB-related members exhibited an obvious response to heat stress. For instance, *BnMRD96, BnMRD111, BnMRD172*, and *BnMRD140* were downregulated, whereas *BnMRD52, BnMRD102*, and *BnMRD177* were upregulated by heat treatment. As shown in the heatmap (Figure 7), the expression patterns of BnMYB-related members varied greatly under abiotic stress conditions. These results implied that *BnMYB-related* genes contribute to a better adaptation of *B. napus* to the natural environment.

**Figure 8:**
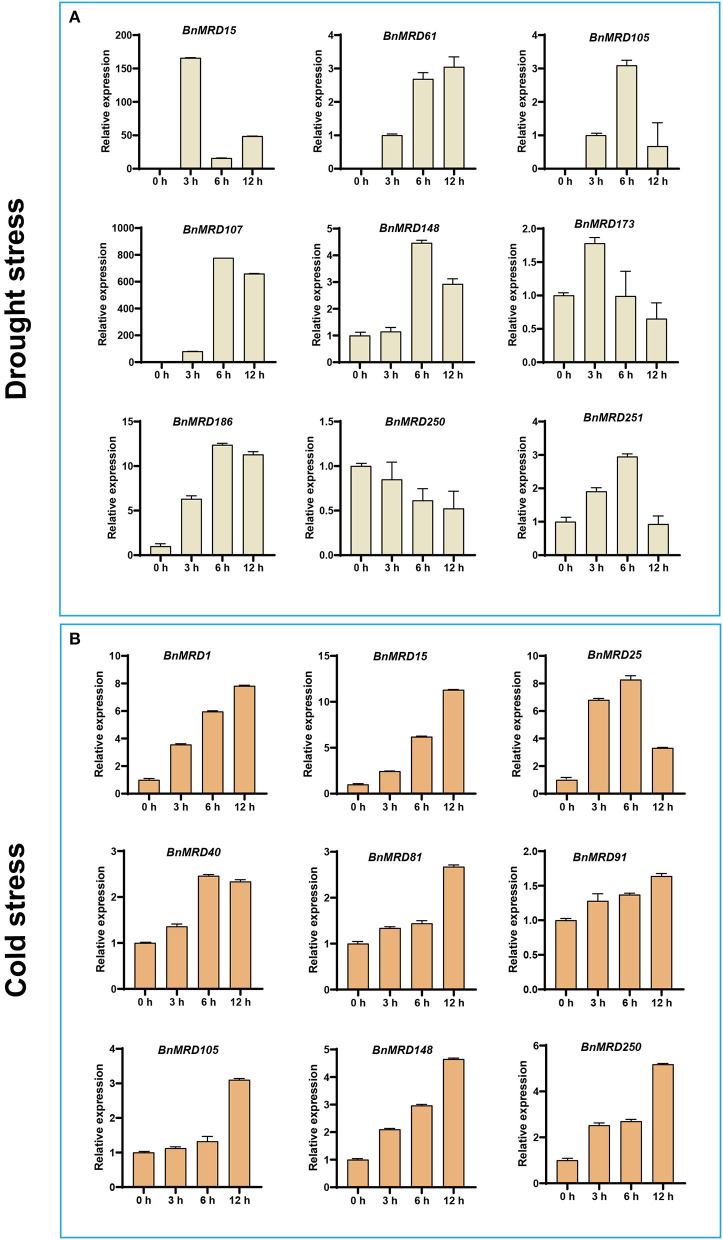
Expression analysis of 13 *BnMYB-related* genes under **(A)** drought treatment and **(B)** cold treatment by qPCR. The expression levels were normalized to *BnActin*. Data represented the mean ± SE (*n* =3).

Considering that quite a few *BnMYB-related* genes exhibited inducible expression under drought or cold stresses, the transcript abundance of nine genes under drought or cold treatment was determined using qPCR (Figure 8). Nine *BnMYB-related* genes including *BnMRD15/25/105/107/148/173/186/250/251*, displayed differential expression under normal and drought stress conditions. Notably, the expression levels of *BnMRD15* and *BnMRD107* were 150 times and 800 times higher after drought treatment for 3 and 6 h than that under normal conditions, respectively (Figure 8A). Moreover, nine genes, including *BnMRD1/15/25/40/81/91/105/148/250*, were upregulated under cold conditions. Except for *BnMRD25*, the transcript abundance of other eight genes elevated gradually from 0 to 12 h of cold treatment. The expression of *BnMRD25* increased by ~7-fold at 3 h of cold treatments compared with that before stress, peaked at 6 h (by 8-fold), and then decreased at 12 h after cold treatment (Figure 8B). These findings provided insights into the potential roles of *BnMYB-related* genes in response to drought and cold stress, and will be helpful to explore candidate *BnMYB-related* genes for further functional determination and stress resistance improvement of *B. napus*.

### *BnMRD107* Enhances Osmotic and Salt Tolerance in Transgenic *B. napus* Seedlings

**Figure 9:**
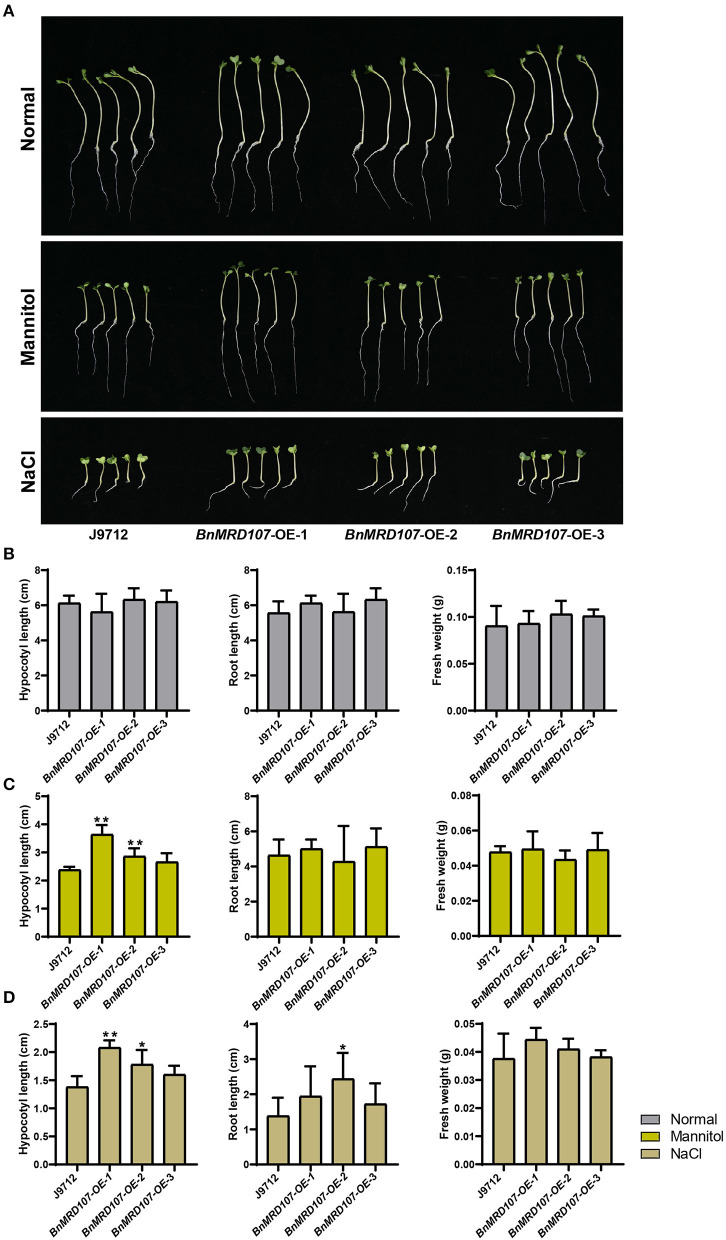
Phenotype of *BnMYBR107*-OE plants under normal conditions, osmotic stress, and salt treatments. **(A)** Growth performance of J9712 and *BnMYBR107*-OE plants under normal conditions, osmotic stress, and salt treatments. **(B–D)** Hypocotyl length, root length, and fresh weight under normal conditions, osmotic stress, and salt treatments. Data are presented as means ± SD (*n* = 5). Asterisks (*) indicate significant differences between the transgenic lines and J9712 based on Duncan's multiple range test (*p < 0.05, **p < 0.01).

**Figure 10:**
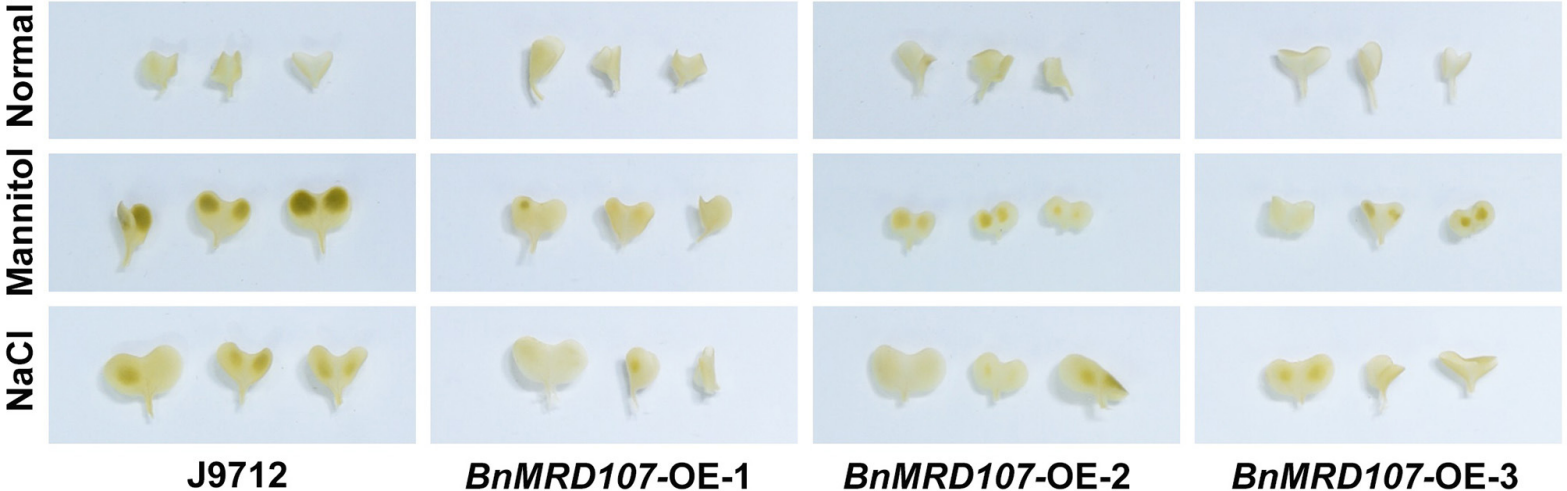
DAB-staining of cotyledons from J9712 and *BnMYBR107*-OE plants under normal conditions, osmotic stress, and salt treatments.

To further investigate the function of *BnMYB-related* genes under abiotic stress in *B. napus*, we carefully scanned the stress-responsive *BnMYB-related* genes. A member belonging to CCA1/R-R-like clade, *BnMRD107*, provoked our interest. *BnMRD107* was significantly induced under drought stress, and its transcript abundance was elevated by almost 800-fold after 6 h of drought stress (Figure 8A). To reveal the potential role of *BnMRD107* in osmotic response of *B. napus*, transgenic rapeseed plants overexpressing *BnMRD107* were obtained by introducing a *BnMRD107*-OE construct into rapeseed using the *Agrobacterium tumefaciens*-mediated transformation approach (Supplementary Figure 3). Three T_2_ generations of homozygous transgenic lines (*BnMRD107*-OE-1, *BnMRD107*-OE-2, and *BnMRD107*-OE-3) were used to evaluate the performance of the plants under osmotic stress and salt treatments, and the wild-type seedlings (J9712) were used as controls (Figure 9). As shown in Figure 9A, no obvious differences were observed between J9712 and *BnMRD107*-OE lines under normal growth conditions. The *BnMRD107*-OE lines showed no significant difference compared with J9712 in hypocotyl length, root length, and fresh weight under normal condition (Figure 9B). However, the *BnMRD107*-OE lines (especially *BnMRD107*-OE-1 and *BnMRD107*-OE-2) showed apparently better growth performance than J9712 plants under both osmotic stress and salt treatments (Figure 9A), and the hypocotyl lengths of *BnMRD107*-OE plants were longer than those of J9712 plants under osmotic and salt stress conditions (Figures 9C,D). To examine the H_2_O_2_ accumulation in *BnMRD107*-OE and J9712 plants under normal, osmotic stress, and salt conditions, DAB-staining analysis of the plant cotyledons was performed. As shown in Figure 10, there was no visible difference in H_2_O_2_ accumulation between the *BnMRD107*-OE and J9712 plants under normal conditions, while *BnMRD107*-OE plants accumulated markedly less H_2_O_2_ than J9712 plants in their cotyledons after osmotic stress and salt treatments. These results indicated that *BnMRD107* conferred osmotic and salt tolerance on transgenic rapeseed seedlings, and the rapeseed plants overexpressing *BnMRD107* might possess stronger ROS-scavenging ability than wild-type J9712 plants.

## Discussion

MYB-related transcription factors comprise a prominent subtype of MYB superfamily in plants (Du et al., 2013). Based on the previous research, there are 64, and 223 *MYB-related* genes in the model plant *Arabidopsis* and *rice*, respectively (Chen et al., 2006, 2019). Recently, 62 genes in *Physcomitrella patens* (Pu et al., 2020), 138 genes in *Solanum tuberosum* (Liu Y. H. et al., 2020), 73 genes in *Musa acuminate*, and 59 genes in *Musa balbisiana* (Tan et al., 2020) were identified as *MYB-related* genes. Plant *MYB-related* genes have been reported to be involved in multiple processes, including cellular and organ morphogenesis, secondary metabolism, circadian rhythm, and drought tolerance (Baldoni et al., 2015). However, there has been little investigation of *MYB-related* genes in *B. napus*, and the functions of BnMYB-related family members are still largely unknown. In the present study, 251 *MYB-related* genes were identified in the *B. napus* genome. The phylogenetic relationships, gene structure, motif composition, syntenic analysis, *cis*-elements, and expression profiles of BnMYB-related family members were investigated systematically.

The present allotetraploid rapeseed (genome AACC) cultivars were generated by interspecific hybridization between its diploid progenitors *B. rapa* (genome AA) and *B. oleracea* (genome AACC) (Chalhoub et al., 2014). MYB-related genes, as a share of the entire MYB family genes, comprise 36.6% (97/265), 44.9% (106/236), 39.8% (202/508), 36.9% (171/464), and 33.9% (251/740) in *Arabidopsis*, rice, *B. oleracea, B. rapa*, and *B. napus*, respectively (Saha et al., 2016; Jin et al., 2017). In this study, 251 *MYB-related* genes were identified in *B. napus*, which showed a significantly higher number than that in the *Arabidopsis* genome (97 genes). In the diploid ancestors (*B. oleracea* and *B. rapa*), 76 and 84 orthologous genes were found, respectively. Four categories of gene duplication events including WGD/segmental duplication, dispersed duplication, proximal duplication, and tandem duplication were detected in *BnMYB-related* genes. Among the 251 sequences, 77.29% (194/251) were implicated in WGD/segmental duplication events, and 20.32% (51/251), 0.01% (3/251), and 0.01% (3/251) involved dispersed duplication, proximal duplication, and tandem duplication, respectively. These results indicated that WGD/segmental duplication and dispersed duplication were the vital driving force for *MYB-related* gene family expansion.

Similar to MYB-related proteins in other plants, BnMYB-related family members were characterized by a single or a partially conserved repeat. In this study, the 251 BnMYB-related proteins were classified phylogenetically into five major clades. CCA1/R-R-like clade is the largest clade, whose members have a common conserved motif SHAQKY(N)F or SHAQK, in which the third W residue was often substituted by A residue. The circadian-related *cis*-acting regulatory elements were found to be enriched in the promoter regions of CCA1/R-R-like clade genes, which were consistent with the result of previous studies, in which CCA1/R-R-like genes were identified as being involved in the circadian rhythm (Lu et al., 2009). A previous study on *Arabidopsis* illustrated that CCA1 protein can bind directly to the promoter of *Light-harvesting chlorophyll a/b protein* (*Lhcb*) to activate its transcription (Lu et al., 2009). Moreover, *cis*-elements associated with auxin response and cell cycle regulation were also found to be generally distributed in the promoter regions of CCA1/R-R-like genes. *Cis*-elements involved in meristem expression were detected in 12 genes including *BnMRD36, BnMRD96*, and *BnMRD130*, and expression analysis showed that these genes exhibited a strong and specific expression in SAM, suggesting their potential roles in meristem development. No consensus motifs were detected in the proteins of I-box-like, CPC-like, and TRF-like clades. However, W residues were evenly distributed in MYB repeats of the members of these clades. CPC-like (II) members contained three W residues in their MYB repeat regions. Investigations on tomato (*Solanum lycopersicum*) revealed that CPC-like members participated in anthocyanin biosynthesis (Ma and Constabel, 2019). Most of the TBP-like proteins had the consensus motif LKDKW(R/K) (N/T). Although the functional analysis of proteins of I-box-like, TRF-like, and TBP-like clades is relatively rare, their expression profile in various tissues and organs suggested that they might play important roles in *B. napus* development. Unlike members of other transcription factor families, the sequence identity of MYB-related proteins was relatively low, which might be relevant to their diverse functions in plants.

Although the MYB-related family widely exists in plant species, there has been much more research into 2R-MYB genes than into *MYB-related* genes in plants. In the present study, we observed that 77.69% (195/251) of *MYB-related* genes were responsive to at least one of the four tested abiotic stresses and ABA treatment, indicating that a large proportion of *MYB-related* genes are involved in the adaptation of *B. napus* to the environment. *Cis*-acting elements are determinate components of the specificity and expression level of genes (Lescot et al., 2002). Five elements associated with stress responsiveness (ARE, WUN-motif, TC-rich repeats, LTR, and DRE) and five hormonal responsive elements (GARE, ABRE, TGA, TGACG-motif, and TCA) are enriched in the promoters of *MYB-related* genes. Drought stress and cold stress are two principal natural disasters that occur during the lifespan of rapeseed. Based on the transcriptome expression data, 184 and 187 *MYB-related* genes showed a transcriptional response to drought and cold stress, respectively. The expression pattern of nine *MYB-related* genes under drought and cold stress, respectively, were verified using qPCR. Five genes, *BnMRD15, BnMRD107, BnMRD148, BnMRD186*, and *BnMRD251*, were strongly induced by drought, which was in accordance with the enrichment of *cis*-elements of MYB binding site involved in drought-inducibility in their promoters. In addition, certain genes (including *BnMRD7, BnMRD9, BnMRD15, BnMRD81*, and *BnMRD102*) that responded to cold stress were verified to contain cold-responsive *cis*-acting element LTRs in their promoters. These genes might be considered as potential targets to improve stress tolerance of *B. napus*.

In a previous study, the expansion of MYB superfamily and hormone-mediated expression of *B. napus* MYB genes were analyzed, and the functions of four 2R-MYB genes in root hair development were studied through ectopic expression in *A. thaliana* (Li et al., 2020). In the present study, comprehensive investigations of MYB-related family members in *B. napus* were performed. The homologs of *MYB-related* genes in eight rapeseed accessions were also identified for evolutionary conservation analysis. Combining *cis-*element analysis and temporospatial and abiotic stress expression profiles, we identified a number of tissue and organ-specific or stress-responsive *MYB-related* genes which might play major roles in *B. napus* development and response to external environmental stimuli. To confirm the function of the stress-responsive *BnMYB-related* genes, we chose *BnMRD107*, which was dramatically upregulated under drought stress, for cloning and transgenic functional verification. Overexpressing *BnMRD107* led to significantly improved resistance to osmotic and salt stresses and enhanced ROS-scavenging ability in rapeseed seedlings. These results suggested that stress-responsive *BnMYB-related* genes, such as *BnMRD107*, can be considered as potential candidate genes for genetic improvement of *B. napus*. Detailed analysis of the candidate genes is required to gain insights into their biological functions and regulatory mechanisms in stress responses of *B. napus*.

## Conclusion

In the current study, we performed a systematic genome-wide analysis of MYB-related family genes in *B. napus*. The 251 identified *BnMYB-related* genes are unevenly located on 19 chromosomes. Phylogenetic- and motif-based classification and diverse temporospatial expression patterns revealed that *BnMYB-related* genes are involved in modulating many growth and development processes in rapeseed. Furthermore, transcriptome data, together with qPCR analysis, illustrated that *BnMYB-related* genes might act as key transcription regulators of the response of *B. napus* to drought and cold stresses. In addition, we identified a gene, *BnMRD107*, which regulates osmotic and salt tolerance positively in *B. napus*. In summary, this study established a comprehensive picture of *MYB-related* gene family in *B. napus*. These findings will not only facilitate in-depth functional investigation of *BnMYB-related* genes but also offer candidate gene resources for improving the stress tolerance of *B. napus*.

## Data Availability Statement

The original contributions generated for the study are publicly available. This data can be found at: NCBI Sequence Read Archive, and the BioProject ID are PRJNA687395 and PRJNA721476.

## Author Contributions

JL and YF conceived the research, designed the experiments, and analyzed the data. JL, KL, SZ, and JW collected the sample materials and performed the experiments. JL prepared the original manuscript. YF and YW revised the manuscript and supervised the project. All authors have read and approved the manuscript.

## Conflict of Interest

The authors declare that the research was conducted in the absence of any commercial or financial relationships that could be construed as a potential conflict of interest.

## Supplementary Material

Supplementary Figure 1 Distribution of MYB-related family genes on *B. napus* chromosomes. Several genes are not shown in this figure because they are located in unassembled scaffolds. Two hundred and thirty-one *MYB-related* genes were assigned to 19 chromosomes, and the names of the chromosomes are indicated at the top of each chromosome. The scale of the chromosomes was in megabases (Mb).

Supplementary Figure 2 Syntenic relationships of *MYB-related* genes between *B. napus* and three closely-allied plant species, *Arabidopsis thaliana* (*A. thaliana*), *Brassica oleracea* (*B. oleracea*), and *Brassica rapa* (*B. rapa*) genomes. Gray lines in the background indicate the collinear blocks within canola and other plant genomes, and the red lines highlight the *MYB-related* gene orthologs.

Supplementary Figure 3 Relative expression level of T_0_ generation of *BnMRD107*-OE transgenic plants.

Supplementary Table 1 List of the primers used in this study.

Supplementary Table 2 General information of *BnMYB-related* genes.

Supplementary Table 3 *Ka/Ks* ratios of the duplicated *BnMYB-related* genes pairs.

Supplementary Table 4 Duplication type of *BnMYB-related* genes.

Supplementary Table 5 *Ka*/*Ks* ratios of the duplicated *MYB-related* gene pairs of *B. napus* and *Arabidopsis*.

Supplementary Table 6 *Ka*/*Ks* calculation of the duplicated *BnMYB-related* gene pairs of *B. napus* and *B. oleracea*.

Supplementary Table 7 *Ka*/*Ks* ratios of the duplicated *BnMYB-related* gene pairs of *B. napus* and *B. rapa*.

Supplementary Table 8 Homologous members of *MYB-related* genes in eight rapeseed accessions.

Supplementary Table 9 *Cis*-elements in the promoter regions of *BnMYB-related* genes.

Supplementary Table 10 FPKM values of *BnMYB-related* genes in tissues/organs at different developmental stages.

Supplementary Table 11 Relative expression of *BnMYB-related* genes under abiotic stresses and ABA treatment.
