# Supplementary figures and images for: Genome-Wide Analysis of Myeloblastosis-Related Genes in Brassica napus L. and Positive Modulation of Osmotic Tolerance by BnMRD107
Source: Front Plant Sci. 2021 Jun 17;12:678202. doi: 10.3389/fpls.2021.678202 (PMC8248502; doi:10.3389/fpls.2021.678202)

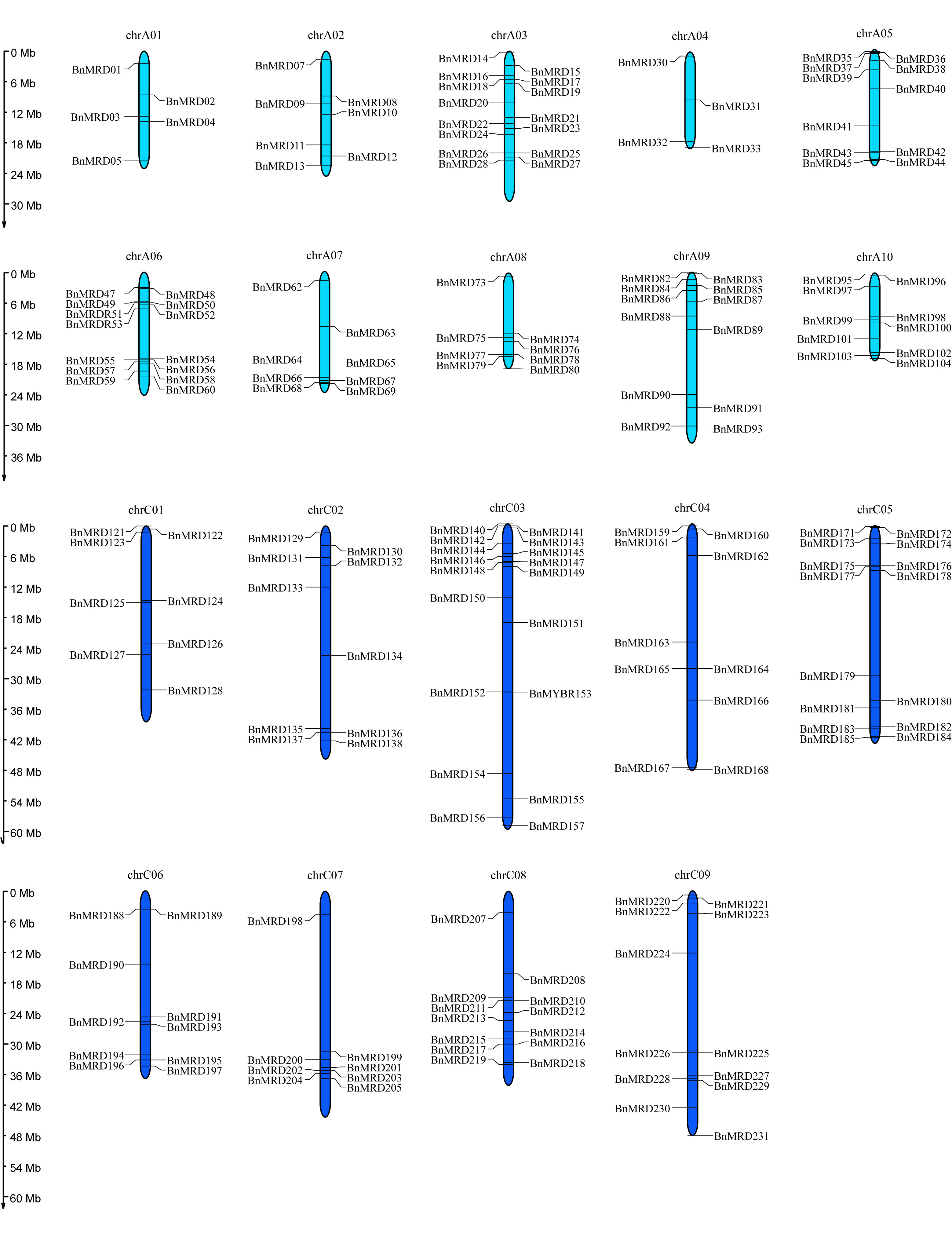

Supplement: Supplementary Figure 1 — Distribution of MYB-related family genes on B. napus chromosomes. Several genes are not shown in this figure because they are located in unassembled scaffolds. Two hundred and thirty-one MYB-related genes were assigned to 19 chromosomes, and the names of the chromosomes are indicated at the top of each chromosome. The scale of the chromosomes was in megabases (Mb). [file Image_1.jpg]

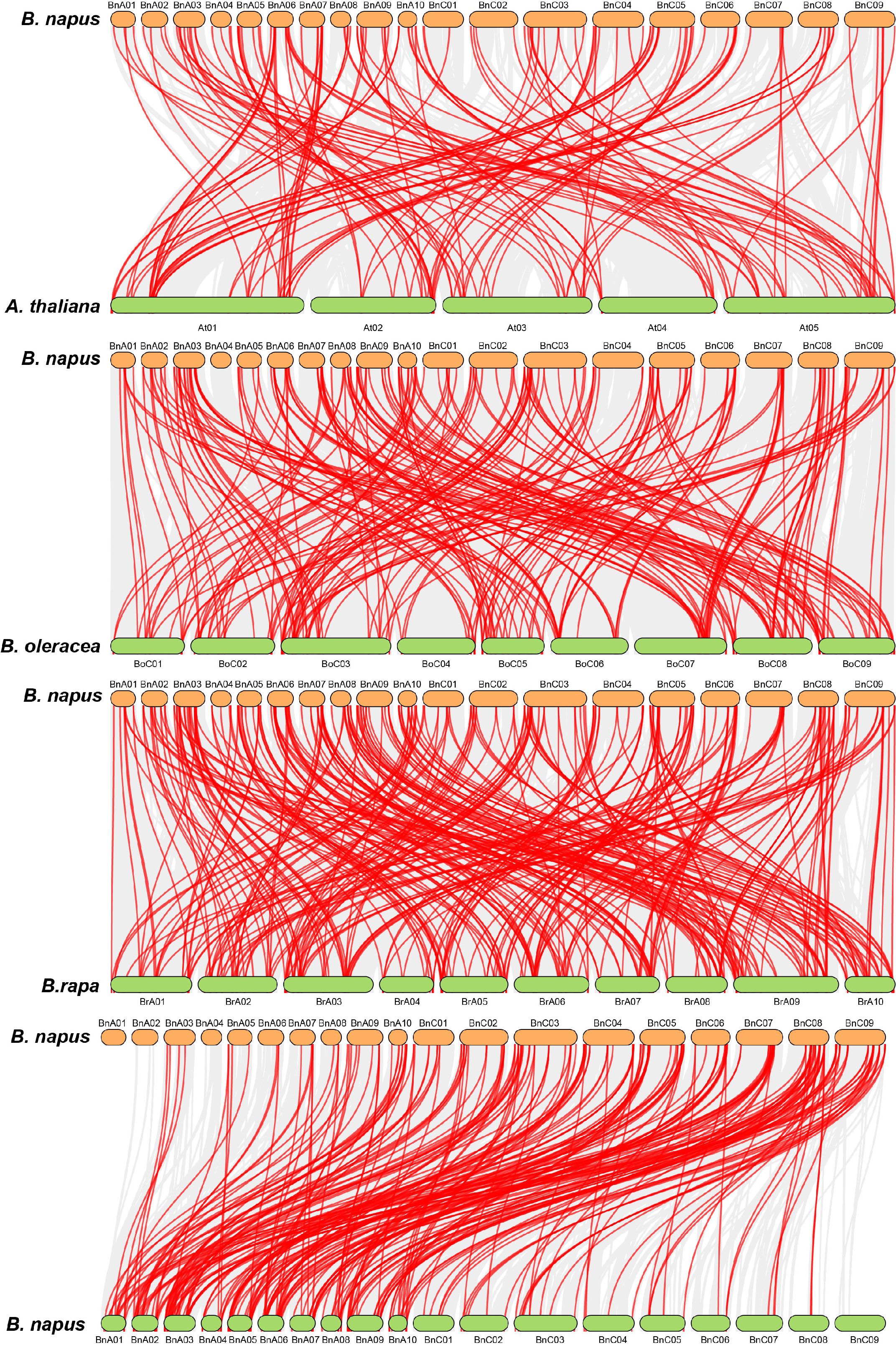

Supplement: Supplementary Figure 2 — Syntenic relationships of MYB-related genes between B. napus and three closely-allied plant species, Arabidopsis thaliana (A. thaliana), Brassica oleracea (B. oleracea), and Brassica rapa (B. rapa) genomes. Gray lines in the background indicate the collinear blocks within canola and other plant genomes, and the red lines highlight the MYB-related gene orthologs. [file Image_2.jpg]

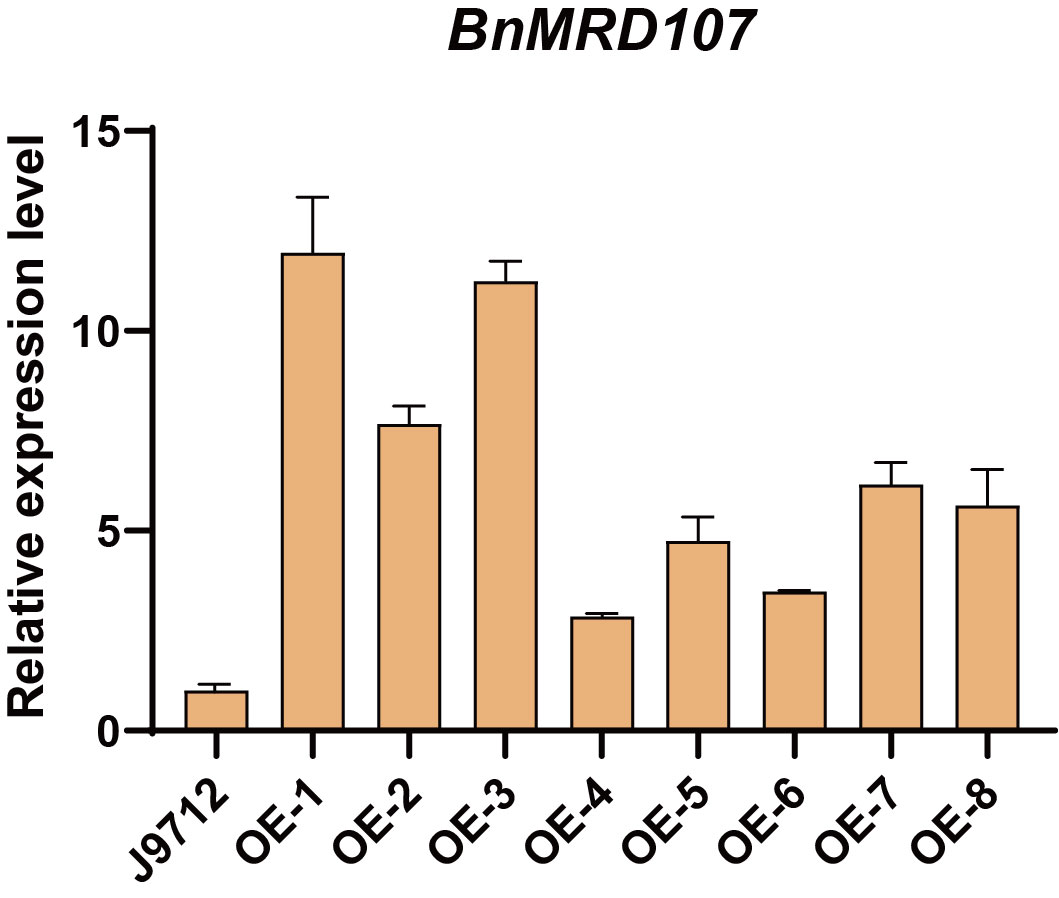

Supplement: Supplementary Figure 3 — Relative expression level of T0 generation of BnMRD107-OE transgenic plants. [file Image_3.jpg]
